# Supplementary material for: Effectiveness of a Lifestyle Change Program on Insulin Resistance in Yaquis Indigenous Populations in Sonora, Mexico: PREVISY
Source: Nutrients. 2023 Jan 23;15(3):597. doi: 10.3390/nu15030597 (PMC9920776; doi:10.3390/nu15030597)
Supplement: Supplementary file 1 [file nutrients-15-00597-s001.zip › nutrients-2176415-supplementary.pdf]

Supplementary Material

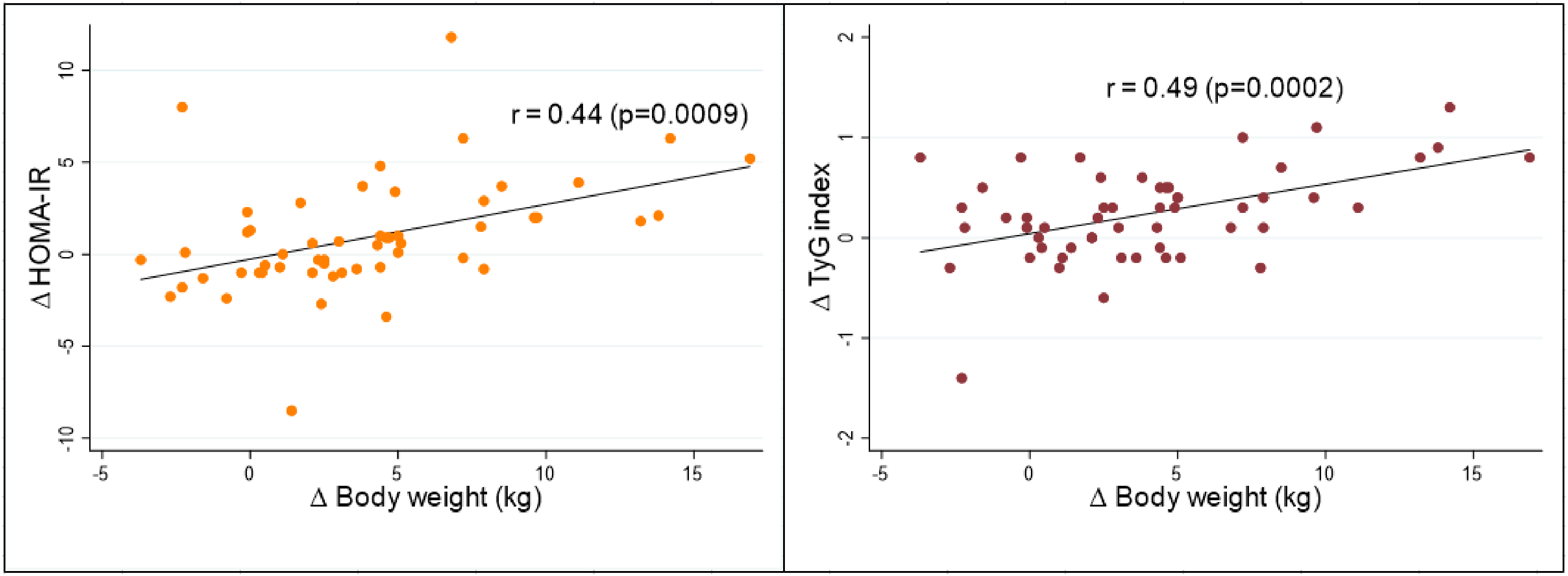

Figure S1. Correlation between the change in body weight and the changes in insulin resistance indicators in the completer subjects in the short term. HOMA-IR: Homeostasis Model Assessment of Insulin Resistance; TyG index: Triglycerides-Glucose Index.

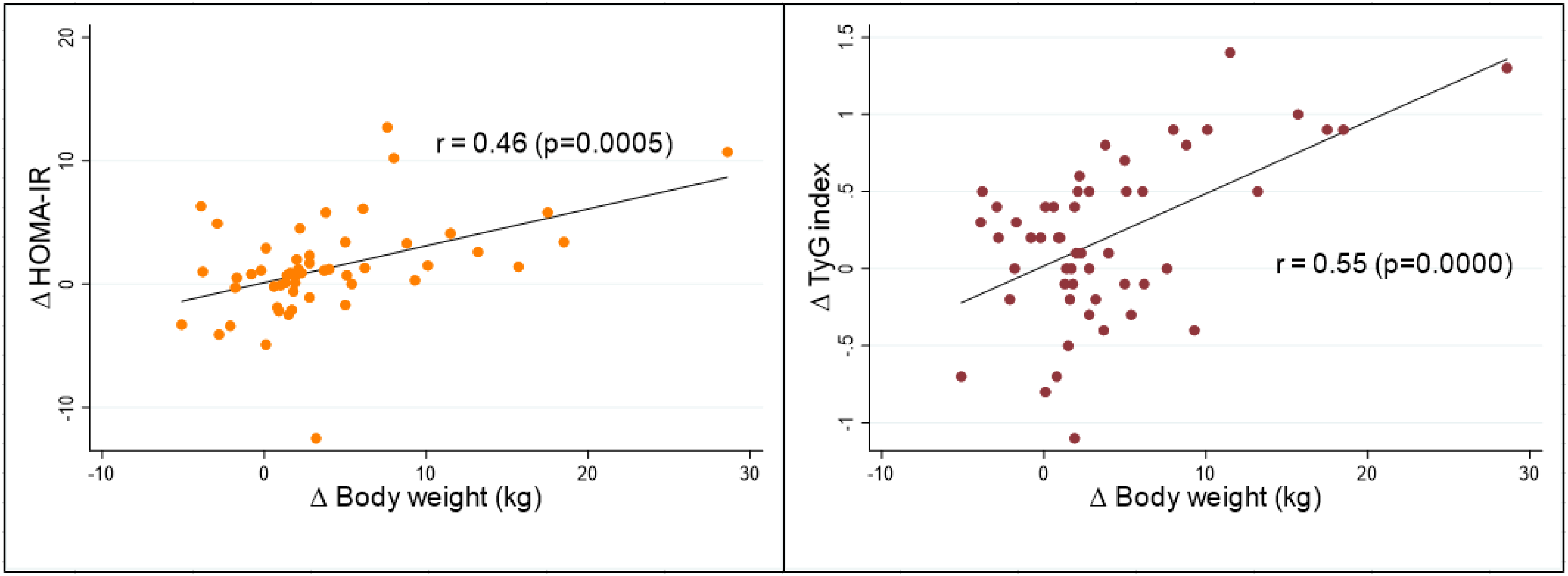

Figure S2. Correlation between the change in body weight and the changes in insulin resistance indicators in the completer subjects in the medium term. HOMA-IR: Homeostasis Model Assessment of Insulin Resistance; TyG index: Triglycerides-Glucose Index.
